# Test-Retest Reliability of Diffusion Measures Extracted Along White Matter Language Fiber Bundles Using HARDI-Based Tractography

**DOI:** 10.3389/fnins.2018.01055

**Published:** 2019-01-14

**Authors:** Mariem Boukadi, Karine Marcotte, Christophe Bedetti, Jean-Christophe Houde, Alex Desautels, Samuel Deslauriers-Gauthier, Marianne Chapleau, Arnaud Boré, Maxime Descoteaux, Simona M. Brambati

**Affiliations:** ^1^Centre de Recherche de l’Institut Universitaire de Gériatrie de Montréal, Montreal, QC, Canada; ^2^Département de Psychologie, Université de Montréal, Montreal, QC, Canada; ^3^Centre de Recherche du CIUSSS du Nord-de-l’île-de-Montréal, Hôpital du Sacré-Cœur de Montréal, Montreal, QC, Canada; ^4^École d’Orthophonie et d’Audiologie, Faculté de Médecine, Université de Montréal, Montreal, QC, Canada; ^5^Sherbrooke Connectivity Imaging Lab, Département d’Informatique, Université de Sherbrooke, Montreal, QC, Canada; ^6^CIUSSS du Nord-de-l’île-de-Montréal, Hôpital du Sacré-Cœur de Montréal, Montreal, QC, Canada; ^7^Université Côte d’Azur, Inria, France

**Keywords:** dMRI, tractography, tractometry, test-retest, language

## Abstract

High angular resolution diffusion imaging (HARDI)-based tractography has been increasingly used in longitudinal studies on white matter macro- and micro-structural changes in the language network during language acquisition and in language impairments. However, test-retest reliability measurements are essential to ascertain that the longitudinal variations observed are not related to data processing. The aims of this study were to determine the reproducibility of the reconstruction of major white matter fiber bundles of the language network using anatomically constrained probabilistic tractography with constrained spherical deconvolution based on HARDI data, as well as to assess the test-retest reliability of diffusion measures extracted along them. Eighteen right-handed participants were scanned twice, one week apart. The arcuate, inferior longitudinal, inferior fronto-occipital, and uncinate fasciculi were reconstructed in the left and right hemispheres and the following diffusion measures were extracted along each tract: fractional anisotropy, mean, axial, and radial diffusivity, number of fiber orientations, mean length of streamlines, and volume. All fiber bundles showed good morphological overlap between the two scanning timepoints and the test-retest reliability of all diffusion measures in most fiber bundles was good to excellent. We thus propose a fairly simple, but robust, HARDI-based tractography pipeline reliable for the longitudinal study of white matter language fiber bundles, which increases its potential applicability to research on the neurobiological mechanisms supporting language.

## Introduction

The characterization of the brain and language network and its development, disruption, and changes over time represents one of the central themes of cognitive neuroscience. Diffusion magnetic resonance imaging (dMRI)-based tractography has been proven to be a valuable tool for the *in vivo* identification of white matter (WM) fiber bundles involved in language and the extraction of measures of their micro- and macro-structural characteristics. However, the ability of this tool to reproduce the same language fiber bundles’ morphology and micro- and macro-structural characteristic measurements when dMRI data is acquired twice from the same participant under the same conditions (i.e., test-retest reliability), has yet to be clearly demonstrated. In fact, while test-retest reliability has already been reported for other neuroimaging techniques that are usually employed in evaluating longitudinal changes in the language brain network (such as resting state and task-based functional MRI, voxel-based morphometry, and cortical thickness; e.g., Jovicich et al., 2009; Zhang et al., 2011; Braun et al., 2012; Birn et al., 2013; Powers et al., 2013; Lin et al., 2015; Seiger et al., 2015; Wang et al., 2016; Madan and Kensinger, 2017; Zhang et al., 2017), test-retest reliability of dMRI-based tractography has received comparatively less attention. This represents a first necessary step to validate the use of this approach in longitudinal studies on language.

It is increasingly accepted that WM associative fiber bundles play a crucial role in mediating the transfer of information among specialized language brain areas, distributed along two main processing streams, namely the dorsal and ventral streams (Hickok and Poeppel, 2000, 2007; Saur et al., 2008; Poeppel et al., 2012; Dick et al., 2014). The central and most widely studied WM fiber bundle of the dorsal stream is the arcuate fasciculus (AF), putatively connecting Broca’s and Wernicke’s territories (Catani and Thiebaut de Schotten, 2012). The AF has been suggested to play a central role in the processing of phonological information and complex syntax in both language production and comprehension (Duffau et al., 2002, 2003; Friederici et al., 2006; Brauer et al., 2011, 2013; Wilson et al., 2011). The major fiber bundles of the ventral stream are the inferior longitudinal fasciculus (ILF), the inferior fronto-occipital fasciculus (IFOF), and the uncinate fasciculus (UF). Their specific contribution to language processing is still a matter of debate. Both the ILF and IFOF are bundles of long association fibers originating in the occipital lobe (Dick et al., 2014). The ILF connects occipital and temporal lobes, while the IFOF connects the occipital and frontal lobes (Catani and Thiebaut de Schotten, 2008; Thiebaut de Schotten et al., 2012; Dick et al., 2014). Both of these bundles have been suggested to play a key role in semantic processing, more specifically in reading and naming (Duffau et al., 2005, 2014; Duffau, 2008; Turken and Dronkers, 2011; Gil-Robles et al., 2013; Han et al., 2013). The UF is a long-range association fiber bundle connecting the anterior temporal lobe with the orbital and polar frontal cortex (Thiebaut de Schotten et al., 2012). While the role of this bundle in language is still controversial, it has been suggested to support semantic retrieval (Lu et al., 2002; Grossman et al., 2004; Catani and Mesulam, 2008) and simple syntactic operations (e.g., processing of phrases) (Friederici et al., 2006).

The use of advanced probabilistic fiber tracking based on high angular resolution diffusion imaging (HARDI) has proven to be particularly suitable for the reconstruction of fiber bundles with complex configurations (i.e., crossing, kissing, or fanning fibers), such as language-related fiber bundles (Alexander et al., 2002; Tuch et al., 2002; Tournier et al., 2012b; Descoteaux, 2015; Farquharson and Tournier, 2016; Jeurissen et al., 2017; Maier-Hein et al., 2017). Up until recently, diffusion tensor imaging (DTI) tractography based on dMRI data has been considered a standard tool for the *in vivo* reconstruction of fiber bundles. However, it has been demonstrated that DTI fails to adequately represent the complex architecture of WM fibers in the brain (Frank, 2001; Alexander et al., 2007; Behrens et al., 2007; Descoteaux et al., 2007, 2009; Jeurissen et al., 2010; Prckovska et al., 2012; Descoteaux, 2015). Standard DTI analysis can represent only one fiber population per voxel, whereas about 66% to 90% of voxels contain a complex fiber configuration (Descoteaux and Deriche, 2008; Jeurissen et al., 2010; Jeurissen et al., 2014). HARDI has been introduced to mitigate some of DTI’s limitations in WM areas with a complex geometry (Alexander et al., 2002; Tuch et al., 2002; Tournier et al., 2012a; Descoteaux, 2015; Farquharson and Tournier, 2016). HARDI measures the diffusion signal along 60 or more gradient directions taken on the sphere in q-space (Descoteaux, 2015). HARDI-based reconstruction techniques such as constrained spherical deconvolution (CSD) aim to estimate the distribution of different fiber orientations within a voxel using a mathematical object known as the fiber orientation distribution function (fODF) (Seunarine and Alexander, 2009). As opposed to the tensor, the fODF allows the estimation of more than one fiber population per voxel, which allows better characterization of WM in regions with a complex architecture (Côté et al., 2013; Descoteaux, 2015).

The combination of micro- and macro-structural measures allows a more comprehensive analysis of WM fiber bundle characteristics. Microstructural properties of bundles reconstructed with tractography are usually inferred from the extraction of different scalar metrics, such as fractional anisotropy (FA), mean diffusivity (MD), radial diffusivity (RD), and axial diffusivity (AD). These measures are sensitive to different fiber properties such as axonal ordering, myelinization, and density (Jones et al., 2013). Although the specific interpretation of these measures is still a matter of debate, they are routinely used in both fundamental and clinical neuroscience studies to provide insights into WM fiber bundles’ profile (Tournier et al., 2012b). The development of CSD based on HARDI data allows the estimation of the number of fiber orientations (NuFO) using the number of local maxima of the fiber orientation distribution (FOD) (Dell’Acqua et al., 2013). NuFO indicates the number of distinct fiber orientations in each voxel, thus providing valuable information on WM complexity. Interestingly, NuFO maps are highly consistent across individuals, which could represent a sensitive marker of age-related changes in WM complexity among healthy populations or changes observed in clinical populations (Dell’Acqua et al., 2013). Macrostructural measures provide complementary information regarding the morphology of the bundles, which includes the volume of the fiber bundles and the mean length of streamlines (MLS) (Girard et al., 2014).

While there is growing interest in the use of tractography and tractometry in longitudinal studies to investigate language-related fiber bundles’ changes over time (e.g., Forkel et al., 2014; Lam et al., 2014; Mandelli et al., 2016; Takeuchi et al., 2016; Asaridou et al., 2017; Chow and Chang, 2017), the test-retest reliability of HARDI-based tractography and tractometry for language-related fiber bundles has yet to be demonstrated. Test-retest reliability refers to the reproducibility of a measure repeated twice for the same participant (Berchtold, 2016). In order for an instrument to be used to detect a change, it has to be able to distinguish between a real change in individuals and a random variation due to the measurement instrument itself (Guyatt et al., 1987). This entails that one of the most crucial aspects to look at when assessing the reliability of a method for longitudinal designs is the test-retest reliability of measurement instruments (Berchtold, 2016). To date, most studies have not integrated reproducibility assessment of their diffusion measures and fiber bundles. This is a critical issue because different factors may affect intra-subject reproducibility such as imaging acquisition parameters (e.g., Jones, 2004; Bisdas et al., 2008; Gao et al., 2009), tractography pipelines (Wang et al., 2012; Kristo et al., 2013; Cousineau et al., 2017), and subject physiological noise (e.g., Pfefferbaum et al., 2003; Farrell et al., 2007). Previous studies have provided evidence of good to excellent test-retest reliability for other methods of analysis of dMRI data, such as tract-based spatial statistics (TBSS), region-of-interest (ROI)-based approaches, and DTI-tractography (Ciccarelli et al., 2003; Heiervang et al., 2006; Danielian et al., 2010; Vollmar et al., 2010; Magnotta et al., 2012; Wang et al., 2012; Cole et al., 2014). However, the test-retest reliability of HARDI-tractography and tractometry has received less attention. Promising evidence of test-retest reliability of this approach comes from the work of Besseling et al. (2012), Kristo et al. (2013) and Cousineau et al. (2017). These studies have demonstrated the overlap of WM fiber bundles reconstructed by means of HARDI-based tractography and the reproducibility of their micro- and macro-structural measures, based on dMRI data obtained from healthy subjects in separate MRI acquisition sessions. Even though these studies were crucial in determining the potential of this approach in longitudinal studies, the only language-related bundle included in all of them is the AF which yielded conflicting results. Thus, the test-retest reliability of HARDI-tractography and tractometry in the main language-related fiber bundles remains to be validated.

In order to fill this gap, the aim of the present study is to assess test-retest reliability of the reconstruction, as well as the micro- and macro-structural characteristics of the major WM fiber bundles associated with language processing reconstructed using probabilistic HARDI-tractography. To this aim, we have collected dMRI data from a sample of healthy individuals at two time-points, one week apart, and reconstructed major WM fiber bundles supporting language functions within the left and right hemispheres (AF, ILF, IFOF, and UF). We expect that no measurable changes in the micro- and macro-structural characteristics of the tracts under study would be observed in that short time period. The test-retest reliability of the fiber bundles’ morphology was obtained by calculating, for each subject, the spatial overlap between each tract’s reconstruction at the two time-points as proposed in Cousineau et al. (2017). Additionally, macro-structural characteristics such as volume and MLS, as well as mean microstructural measures such as the tensor-based metrics FA, MD, RD, AD, and the FOD-based measure NuFO (Dell’Acqua et al., 2013) were extracted for each bundle and their reproducibility was assessed.

## Materials and Methods

### Participants

Eighteen right-handed cognitively unimpaired participants (age: *M* = 64.61 y.o. ± 7.99; education: *M* = 16.16, ± 3.42 years; 9 women, 9 men) with no history of psychiatric or neurological conditions were scanned at two time-points, one week apart. The study was approved by the research ethics committee of the Center intégré universitaire de santé et de services sociaux du Nord-de-l’Ile-de Montréal (Project #MP-32-2018-1478) and written informed consent was obtained from all participants.

### Image Acquisition

The diffusion MRI protocol was acquired using a Skyra 3T MRI scanner (Siemens Healthcare, United States) at the radiology department of Hôpital du Sacré-Coeur of Montreal. At each of the two scanning occasions participants underwent the same acquisition sequence. One high resolution 3D T1-weighted (T1w) image (TR = 2200 ms, TE = 2.96 ms, TI = 900 ms, FOV = 250 mm, voxel size = 1 mm × 1 mm × 1 mm, matrix = 256 × 256, 192 slices, flip-angle = 8) was acquired using a Magnetization Prepared Rapid Gradient Echo (MP-RAGE) sequence. A diffusion weighted imaging (DWI) sequence was also acquired (TR = 8051 ms, TE = 86 ms, FOV = 230 mm, voxel size = 2 mm × 2 mm × 2 mm, flip angle = 90°, bandwidth = 1698; EPI factor = 67; 68 slices in transverse orientation) with one image (*b* = 0 s/mm^2^) and 64 images with non-collinear diffusion gradients (*b* = 1,000 s/mm^2^) in a posterior-anterior (PA) acquisition, as well as two additional images (*b* = 0 s/mm^2^): one in a PA acquisition, namely in the same direction as the diffusion gradients, and the other in an anterior-posterior (AP) acquisition, namely in the opposite direction of the diffusion gradients.

### dMRI Data Analysis

All analysis steps were conducted using the Toolkit for Analysis in Diffusion MRI (TOAD) pipeline^1^.

#### Pre-processing

Pre-processing steps included denoising, motion/eddy/distortion corrections, upsampling, registration, segmentation and parcellation, and masking. First, DWI was noise-corrected using overcomplete local principal component analysis (PCA) using the Matlab toolbox DWI Denoising Software (Manjo et al., 2013) The FMRIB Diffusion toolbox EDDY of FSL 5.0.11 (publicly available neuroimaging software^2^) (Jenkinson et al., 2012) was used to correct all images for subject movement, eddy-currents, and susceptibility-induced distortions using AP-PA images. Gradient directions were corrected corresponding to motion correction parameters (motion for each subject at each timepoint is reported in the Supplementary Material). T1w images were processed with Freesurfer’s pipeline 6.0.0 (Dale et al., 1999; Desikan et al., 2006) for segmentation and parcellation of gray and WM into anatomical regions. DWI was upsampled to 1 mm isotropic resolution using a trilinear interpolation (Girard and Descoteaux, 2012; Raffelt et al., 2012; Smith et al., 2012; Tournier et al., 2012a; Dyrby et al., 2014) and the segmented and parcellated T1w was registered to the DWI using FMRIB’s linear registration tool (FLIRT) from FSL. This step allowed us to carry out anatomically constrained tracking (ACT) (see next section for further details). Finally, a mask image was obtained from the segmented T1w image and served to seed streamlines on the gray matter-white matter interface (Tournier et al., 2012a).

#### Tractography

Fiber orientation distribution functions (fODFs) were estimated using CSD. A whole-brain tractogram was computed using MRtrix3’s probabilistic tractography algorithm with ACT^3^ (Tournier et al., 2012a). ACT uses the T1w (i.e., the segmented anatomical image obtained from Freesurfer) to limit potential false-negatives (i.e., no-connections) and improve WM coverage in general (Guevara et al., 2011; Girard and Descoteaux, 2012; Smith et al., 2012; Girard et al., 2014; Mori and Tournier, 2014). The AF, ILF, IFOF, and UF were reconstructed from the tractogram using the White Matter Query Language (WMQL) (Wassermann et al., 2016). WMQL is a user-friendly method to carry out WM bundle extraction from tractography in a nearly automatic way. It allows us to consistently define bundles across subjects without manually specifying regions of interest. It consists in writing queries with the WMQ language describing the WM bundles to be reconstructed using anatomical definitions from Freesurfer’s Desikan-Killiany atlas. In order to be able to extract the fiber bundles using the written queries, Freesurfer’s gray and WM parcellation was overlaid on the tractogram. The queries were then automatically interpreted by tractography tools. The queries used to reconstruct the fiber bundles are presented in the Supplementary Material. Outlier streamlines were then removed from each tract using a tract-filtering algorithm (Côté et al., 2015). The following diffusion and bundle measures were extracted along each fiber bundle for each participant: FA, MD, AD, RD, NuFO (Dell’Acqua et al., 2013), volume, and MLS. All tractography steps were performed in native space since non-linear normalization with diffusion MRI data requires local reorientations and warping which affects the gradient table at every voxel (bval/bvec) (Vollmar et al., 2010). Bringing the T1-w image into native diffusion space with linear affine registration and using the Freesurfer parcellation in this space is more robust (Girard et al., 2014, 2015).

### Statistical Analysis

Test-retest analyses were carried out in two steps. First, we used the weighted dice similarity coefficient (wDSC) to determine the degree of overlap between the reconstructed fiber bundles at Times 1 and 2 as in Cousineau et al. (2017). DSC is a statistical metric that ranges between 0 and 1 and is used to assess the degree of overlap between two volumes (Dice, 1945). The wDSC is a variation of this metric and gives more weight to voxels with more streamlines. This is important to take into account considering the fact that WM bundles have more streamlines in their middle than in the extreme portions (Cousineau et al., 2017). In the two previous studies which used this measure to assess the test-retest reliability of CSD-based reconstruction of WM tracts (Besseling et al., 2012; Cousineau et al., 2017), the minimum value of Dice was 0.70. Therefore, this value was used as the acceptable threshold for a good wDSC in our study. The wDSC was computed using the following formula from Cousineau et al. (2017):

$$D(W_{i},\;W_{j})=\frac{\Sigma_{v^{\prime }}\;W_{i,v^{\prime }}+\Sigma_{v^{\prime }}\;W_{j,v^{\prime }}}{\Sigma_{v}\;W_{i,v^{\prime }}+\Sigma_{v}\;W_{j,v}}$$

where *Wi* and *Wj*, respectively, represent the bundles at Time 1 and Time 2, and *v*′ represents the voxels from the two reconstructions of the bundles (*Wi* and *Wj*) that overlap.

To do so, T1-weighted images in diffusion space taken at Time 1 were registered linearly to anatomical images taken at Time 2 (i.e., seven days later) for each subject with Advanced Normalization Tools (ANTs), version ≥ 2.1 (Tustison et al., 2014)^4^. Transformation matrices were applied to all Time 1 bundles using TractQuerier’s tract_math tool (Wassermann et al., 2016). Once the two fiber bundles of each subject were in the same space, wDSCs were computed with the tractometry pipeline from the Sherbrooke Connectivity Imaging Lab (SCIL) http://scil.dinf.usherbrooke.ca/?lang=fr. The right UF bundle could not be reconstructed in one participant. Analyses were therefore conducted with a sample of 17 participants for that bundle.

In a second step, we combined two complementary analyses, the intra-class correlation coefficient (ICC) and the Bland-Altman plots to assess the test-retest reliability of each of the measures extracted in each reconstructed fiber bundle. The intra-class correlation coefficient (ICC) (Shrout and Fleiss, 1979; McGraw and Wong, 1996) is a widely used statistical approach to assess agreement in test-retest reliability studies in different fields, including neuroimaging (e.g., Zhang et al., 2011; Braun et al., 2012; Birn et al., 2013; Duda et al., 2014; Duan et al., 2015). The ICC is calculated from an analysis of variance and can be broadly defined as the ratio of between-subject variance to the total variance (including within-subject variance and residue) (Berchtold, 2016). ICC values range from 0 to 1 and can be categorized into four levels of test-retest reliability: excellent (ICC > 0.75), good (ICC = 0.60 to 0.74), fair (ICC = 0.40 to 0.59), and poor (ICC < 0.40) (Fleiss, 2003). ICC estimates and their 95% confidence intervals were calculated using SPSS version 25 based on a single measurement, absolute-agreement, two-way mixed-effects model. The formula used for computing this ICC (McGraw and Wong, 1996) is as follows:

$$\frac{{\mathrm{MS}}_{R}-{\mathrm{MS}}_{E}}{{\mathrm{MS}}_{R}+(k-1){\mathrm{MS}}_{E}+\frac{k}{n}({\mathrm{MS}}_{C}-{\mathrm{MS}}_{E})}$$

where MS_*R*_ is the mean square for rows, MS_*C*_ is the mean square of columns, MS_*E*_ is the mean square for error, *k* is the number of measurements, and *n* is the number of subjects.

We also created Bland and Altman plots which provide a visual assessment of the agreement of the two time-points (test and retest) of each measure in all four fiber bundles bilaterally (Bland and Altman, 1999). The created graphs are scatter plots with the Y axis representing the difference between the measurements at the two timepoints and the X axis representing the mean of these measures. Good agreement between measurements at two time-points exists if 95% of the data falls within ± 2 standard-deviations of the mean of differences.

## Results

**FIGURE 1:**
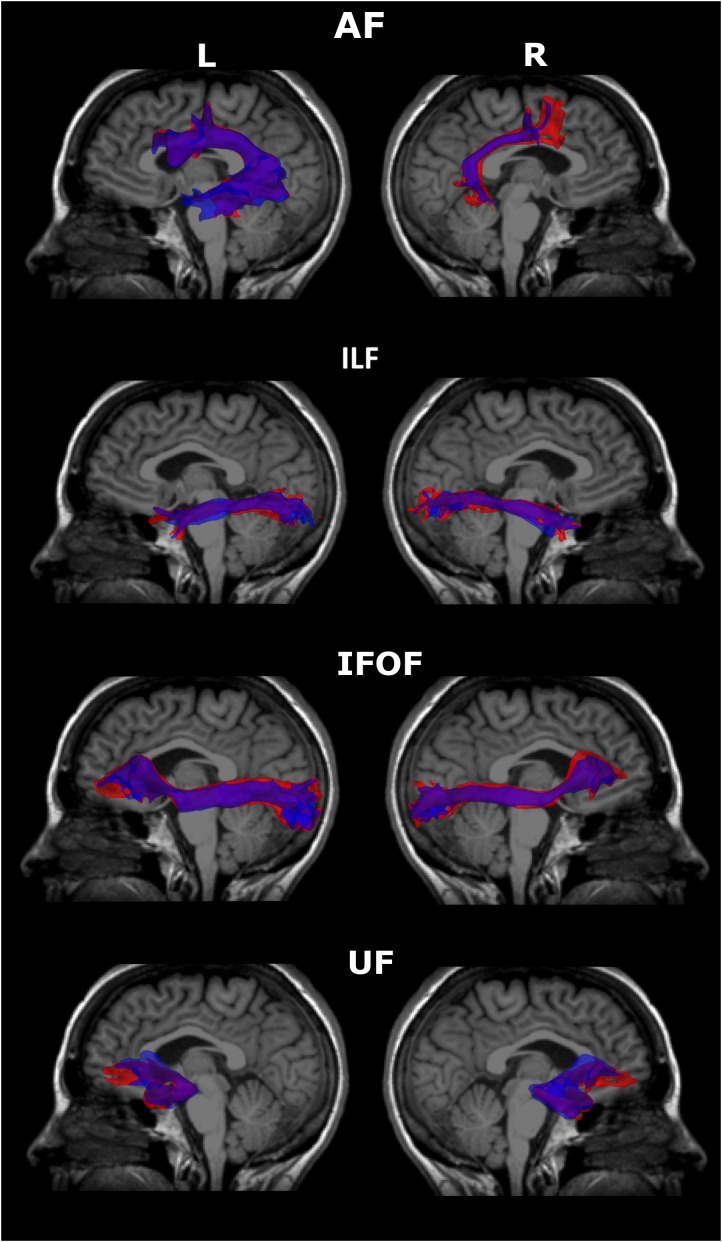
Overlapped 3D volume representations of the reconstructed fiber bundles at the two scanning time-points in a representative subject. Please note that these do not reflect the wDSC values. Blue = time 1, red = time 2, purple indicates the overlap. AF, arcuate fasciculus; ILF, inferior longitudinal fasciculus, IFOF, inferior fronto-occipital fasciculus; uncinate fasciculus; L, Left; R, Right.

The degree of overlap was good for all four reconstructed fiber bundles (AF, ILF, IFOF, and UF, bilaterally) between Time 1 and Time 2, with wDSC values ranging between 0.71 and 0.87 (values for each fiber bundle are reported in Table 1). Figure 1 illustrates the bundle overlap for a representative subject. One must note that the figure reflects the *raw* bundle overlap rather than the weighted overlap represented by the wDSC which gives more weight to voxels with more streamlines.

**Table 1:** wDSC values, ICC estimates and their 95% confidence intervals for all measures and fiber bundles.

	wDSC	FA		AD		MD		RD		NuFO		Volume		MLS	
		ICC	CI	ICC	CI	ICC	CI	ICC	CI	ICC	CI	ICC	CI	ICC	CI
*AF*															
Left	0.86	0.89^∗∗∗^	0.61–0.96	0.91^∗∗∗^	0.78–0.97	0.91^∗∗∗^	0.79–0.97	0.92^∗∗∗^	0.81–0.97	0.62^∗∗^	0.22–0.84	0.83^∗∗∗^	0.61–0.93	0.71^∗∗∗^	0.37–0.88
Right	0.83	0.85^∗∗∗^	0.65–0.94	0.71^∗∗∗^	0.38–0.88	0.86^∗∗∗^	0.67–0.95	0.89^∗∗∗^	0.73–0.96	0.68^∗∗^	0.33–0.87	0.79^∗∗∗^	0.52–0.92	0.87^∗∗∗^	0.68–0.95
*ILF*															
Left	0.79	0.78^∗∗∗^	0.52–0.91	0.95^∗∗∗^	0.86–0.98	0.95^∗∗∗^	0.86–0.98	0.88^∗∗∗^	0.70–0.95	0.50^∗^	0.05–0.78	0.79^∗∗∗^	0.53–0.91	0.84^∗∗∗^	0.62–0.94
Right	0.71	0.86^∗∗∗^	0.67–0.94	0.88^∗∗∗^	0.70–0.95	0.90^∗∗∗^	0.75–0.96	0.89^∗∗∗^	0.73–0.96	0.56^∗∗^	0.14–0.81	0.75^∗∗∗^	0.46–0.90	0.89^∗∗∗^	0.65–0.96
*IFOF*															
Left	0.84	0.87^∗∗∗^	0.70–0.95	0.89^∗∗∗^	0.72–0.96	0.84^∗∗∗^	0.62–0.94	0.83^∗∗∗^	0.60–0.93	0.62^∗∗^	0.22–0.84	0.70^∗∗∗^	0.14–0.90	0.70^∗∗^	0.35–0.88
Right	0.87	0.85^∗∗∗^	0.65–0.94	0.92^∗∗∗^	0.79–0.97	0.86^∗∗∗^	0.66–0.94	0.84^∗∗∗^	0.62–0.94	0.63^∗∗^	0.25–0.85	0.58^∗∗^	0.12–0.83	0.69^∗∗^	0.33–0.87
*UF*															
Left	0.78	0.62^∗∗^	0.22–0.84	0.85^∗∗∗^	0.65–0.94	0.83^∗∗∗^	0.60–0.93	0.76^∗∗∗^	0.45–0.90	0.61^∗∗^	0.21–0.83	0.71^∗∗∗^	0.38–0.88	0.68^∗∗^	0.33–0.87
Right	0.83	0.74^∗∗∗^	0.42–0.90	0.82^∗∗∗^	0.58–0.93	0.80^∗∗∗^	0.52–0.92	0.77^∗∗∗^	0.48–91	0.69^∗∗^	0.32–0.87	0.41^∗^	−0.08–0.74	0.82^∗∗∗^	0.56–0.93

*AF, arcuate fasciculus; ILF, inferior longitudinal fasciculus; IFOF, inferior fronto-occipital fasciculus; UF, uncinate fasciculus; FA, fractional anisotropy; AD, axial diffusivity; MD, medial diffusivity; RD, radial diffusivity; NuFO, number of fiber orientations; MLS, mean length of streamlines; wDSC, weighted dice similarity coefficient; ICC, Intra-class correlation coefficient estimates; CI, 95% confidence intervals of the ICC. ^∗^p < 0.05; ^∗∗^p < 0.01; ^∗∗∗^p < 0.001.*

Table 1 shows the ICC estimates, their 95% confidence intervals, and *p* values of the diffusion measures of interest, namely FA, MD, RD, AD, NuFO, volume, and MLS. FA, AD, MD, RD, and MLS measures showed consistently good to excellent test-retest reliability (ICCs = 0.62–0.95) across all four WM fiber bundles, bilaterally. Volume showed fair reliability in the right IFOF and UF (ICC = 0.41–0.58), and good to excellent reliability in all other bundles. NuFO showed the lowest reliability; test-retest reliability was fair in the ILF bilaterally and good in all other bundles.

**FIGURE 2:**
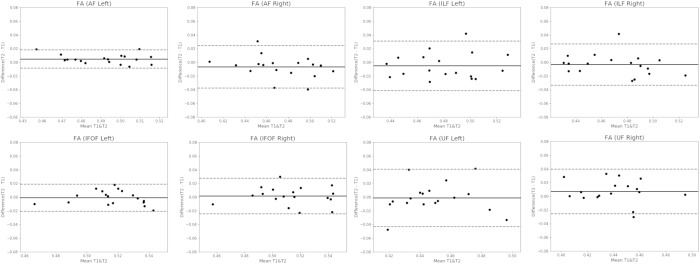
Bland-Altman Plots for the FA metric in all four fiber bundles, bilaterally. The Y axis represents the mean difference between the measurements at the two timepoints and the X axis represents the mean of these measures. The upper and lower dashed lines represent the two limits of agreements at ± 2 standard-deviations of the mean of differences (i.e., the 95% confidence interval). The solid line represents the mean of the differences between the two timepoints. The dots represent the individual subjects. FA, fractional anisotropy; AF, arcuate fasciculus; ILF, inferior longitudinal fasciculus; IFOF, inferior fronto-occipital fasciculus; UF, uncinate fasciculus; T1, time 1; T2, time 2.

In Figure 2, we only present the Bland and Altman plots created for the FA measure for the sake of brevity and clarity. Bland–Altman analysis showed high reproducibility (95% CI = 0.019, −0.01 for the left AF; 0.025, −0.04 for the right AF; 0.03, −0.04 for the left ILF; 0.03, −0.03 for the right ILF; 0.02, −0.02 for the left IFOF; 0.03, −0.02 for the right IFOF; 0.04, −0.04 for the left UF; and 0.2, −0.2 for the right UF) with little difference (mean difference = 0.005 for the left AF, −0.006 for the right AF, −0.004 for the left ILF, −0.003 for the right ILF, −0.0008 for the left IFOF, 0.002 for the right IFOF, 0.001 for the left UF, and 0.03 for the right UF). A total of 88% (right AF, left and right UF) to 94% (Left AF, left and right ILF, left and right IFOF) of data points were within these limits. The 6 other plots are reported in the Supplementary Material. All plots were consistent with the ICC analyses.

## Discussion

The aim of this study was to demonstrate the test-retest reliability of the reconstruction and micro- and macro-structural characteristics of major WM language fiber bundles using probabilistic CSD-tractography based on HARDI data. The dMRI data were obtained on a group of healthy subjects at two timepoints, spanning one week. First, the results demonstrated that all the reconstructed fiber bundles have a good overlap between the two timepoints. Secondly, tract-specific measures usually used in studying microstructural WM characteristics, such as FA, MD, RD, and AD, as well as the macrostructural measure MLS showed good to excellent test-retest reliability in the AF, ILF, IFOF, and UF, bilaterally. Volume, another macrostructural property, showed good to excellent reproducibility for some fiber bundles (AF, ILF, as well as the IFOF, and UF in the left hemisphere) but only fair reproducibility for others (IFOF and UF in the right hemisphere). NuFO showed good test-retest reliability for all fiber bundles, except the ILF which showed only fair test-retest reliability. Our results agree with and, at the same time, critically expand on previous studies that investigated the test-retest reliability of probabilistic CSD-tractography (Besseling et al., 2012; Cousineau et al., 2017). These results represent a first necessary validation protocol for longitudinal studies in research in the cognitive neuroscience of language. Assessing test-retest reliability of the reconstruction of fiber bundles and of their micro- and macro-structural measures is of paramount importance for the use of this approach in longitudinal studies, as it allows to ascertain that the observed variations truly reflect the changes that may take place in WM over time and are not due to the variability inherent to dMRI data processing, instead (Cousineau et al., 2017).

Diffusion MRI tractography is presently the only method that allows the reconstruction of WM fiber bundles *in vivo*. For this reason, in the last decades it has gained tremendous popularity in the field of neuroscience and its potential to map the human connectome is widely recognized. In the last years, an increasing number of big data initiatives has been developed in order to collect longitudinal dMRI data in healthy individuals with the ultimate goal to describe the changes of dMRI over the lifespan and to link these changes to cognitive performance (Howell et al., 2017). In order to fully benefit from the potential of longitudinal dMRI data, it is necessary to demonstrate the test-retest reliability of dMRI-based tractography. The present work provides critical information to investigate two important questions. When we obtain dMRI data in two separate acquisition sessions one week apart in the same subjects, using probabilistic CSD-tractography with ACT and tractometry based on HARDI data, can we 1) reconstruct overlapping WM language fiber bundles? and 2) extract similar micro-and macro-structural measure values?

Regarding the first question, our data seem to provide an affirmative response. The obtained wDSC values determining the degree of overlap of the bundles reconstructed at the two timepoints using probabilistic CSD-tractography ranged between 0.71 and 0.87. Based on the minimum value (0.70) of Dice found in the two studies which used this metric to assess the test-retest reliability of CSD-based reconstruction of WM tracts (Besseling et al., 2012; Cousineau et al., 2017), our wDSC values indicate that all the fiber bundles investigated in the present study have good test-retest reliability. In addition, our wDSC values are consistent with the values obtained in previous studies aimed at validating test-retest reliability of probabilistic CSD-tractography in other fiber bundles, such as the cingulum, optic radiation, and the corpus callosum (Besseling et al., 2012; Cousineau et al., 2017). We also report excellent overlap for the AF and IFOF, which is consistent with Cousineau et al. (2017) who used a similar tractography pipeline. The overlap obtained in our study is greater than what has been reported by Besseling et al. (2012) in which, in order to reconstruct the AF, they only used seed and target ROIs. Considering the complex anatomy of the AF, a tractography method allowing the use of more specific anatomical priors, as we did in the present study, might improve the reconstruction of this complex tract and thus allow for a better reproducibility of its morphology.

Our study also confirms the reproducibility of tensor metrics and MLS. Test-retest reliability of tensor metrics (FA, MD, RD, and AD) has been previously studied using DTI-based tractography (Ciccarelli et al., 2003; Heiervang et al., 2006; Danielian et al., 2010; Vollmar et al., 2010; Wang et al., 2012; Buchanan et al., 2014). However, studies using this approach have not always reported satisfactory results. For example, in one study, poor test-retest reliability was observed with AD across all studied fiber bundles (Danielian et al., 2010), whereas others reported tract-specific variability of the reproducibility of FA and MD (Wang et al., 2012). There are several sources of variability in diffusion MRI that can affect the test-retest reliability of tractography or the measures of WM structural characteristics (Danielian et al., 2010). These include, but are not limited to, partial volume effects introduced by the DTI model, bad anatomical priors, as well as potential inter- and intra-rater reliability of ROI placement in seed-based approaches for tractography (Wakana et al., 2007; Danielian et al., 2010; Cousineau et al., 2017). In the present study, we used an approach that attempts to reduce variability from these sources by using HARDI-based state-of-the-art tracking algorithms based on ACT and probabilistic tracking algorithms which have the potential to yield fuller, longer bundles that better reach the cortex (Mori and Tournier, 2014; Maier-Hein et al., 2017), novel approaches to extract the bundles from the tractogram (i.e., WMQL), as well as good anatomical priors (Conturo et al., 1999; Catani et al., 2002; Hagmann et al., 2003; Huang et al., 2004; Wakana et al., 2007). Using this approach, we were able to demonstrate good to excellent reliability of all tensor-based metrics which are the microstructural measures most commonly used in dMRI studies on language, and MLS, a macrostructural measure, in all language fiber bundles. This represents an important step towards the validation of this approach in the longitudinal study of language fiber bundles. On the other hand, the test-retest reliability of NuFO was less than good in some tracts. To the best of our knowledge, no previous study has investigated the test-retest reliability of this measure. Our results seem to encourage further longitudinal validation of this measure before adopting it in longitudinal studies. Additionally, our results for the volume, another macrostructural measure, were consistent with previous studies that reported inconsistent test-retest reliability for this measure across fiber bundles, using probabilistic CSD-tractography (Besseling et al., 2012) or DTI-based tractography (Wang et al., 2012).

Even though the present results are very promising, particularly for the tensor metrics and MLS, future studies should be designed in order to confirm our findings. First, these results should be reproduced in larger groups. Secondly, the use of CSD allows to resolve multiple fiber orientations at reasonable angles with a properly data-driven response function at lower *b*-values and 64 directions as in the present study (Tournier et al., 2007; Descoteaux et al., 2009; Raffelt et al., 2012). Nevertheless, utilizing multi-*b*-value sequences, such as *b* = 1000 s/mm^2^, *b* = 2000 s/mm^2^, *b* = 3000 s/mm^2^, or *b* = 1000 s/mm^2^, and *b* = 3000 s/mm^2^, could help to interpret the differences obtained in the present study by considering other available measures, such as intracellular, extracellular, and isotropic volume (Raffelt et al., 2012).

## Conclusion

In conclusion, in an era where initiatives to collect dMRI longitudinal data are multiplying and fiber tracking is considered one of the most popular tools to follow changes in the language network over time, the question of test-retest reliability of dMRI tractography is of paramount importance. Our study provides critical evidence indicating the test-retest reliability of probabilistic CSD-tractography. As in previous studies which demonstrated test-retest reliability of TBSS or DTI-tractography (e.g., Kitamura et al., 2013; Forkel et al., 2014; Poudel et al., 2015), the present results support the use of probabilistic CSD-tractography to study language fiber bundles in longitudinal studies in healthy and clinical populations interested in language related fiber bundles.

## Author Contributions

MB drafted the manuscript, contributed to the design of the study, reviewed the literature, and collected, analyzed, and interpreted the data. SB and KM designed the study, contributed to data collection, supervised data analysis and interpretation, and contributed to the drafting of the manuscript. MD contributed to the design of the study and to the development of the tractography pipeline. AD contributed to the design of the study and MRI data acquisition. CB and MC helped with the data analysis. CB, MD, AB, J-CH, and SD-G developed the tractography pipeline. All authors revised the final version of the manuscript.

## Conflict of Interest Statement

The authors declare that the research was conducted in the absence of any commercial or financial relationships that could be construed as a potential conflict of interest.

## Footnotes

1 http://www.unf-montreal.ca/toad

2 http://www.fmrib.ox.ac.uk/fsl/

3 https://github.com/jdtournier/

4 http://stnava.github.io/ANTs/
