# Supplementary material for: Test-Retest Reliability of Diffusion Measures Extracted Along White Matter Language Fiber Bundles Using HARDI-Based Tractography
Source: Front Neurosci. 2019 Jan 14;12:1055. doi: 10.3389/fnins.2018.01055 (PMC6339903; doi:10.3389/fnins.2018.01055)
Supplement: Supplementary file 1 [file Image_1.pdf]

Supplementary Material

Test-retest reliability of diffusion measures extracted along white matter language fiber bundles using HARDI-based tractography

Mariem Boukadi, Karine Marcotte, Christophe Bedetti, Jean-Christophe Houde, Alex Desautels, Samuel Deslauriers-Gauthier, Marianne Chapleau, Arnaud Boré, Maxime Descoteaux, & Simona M. Brambati*

*** Correspondence:** Simona M. Brambati: simona.maria.brambati@umontreal.ca

Supplementary Figures

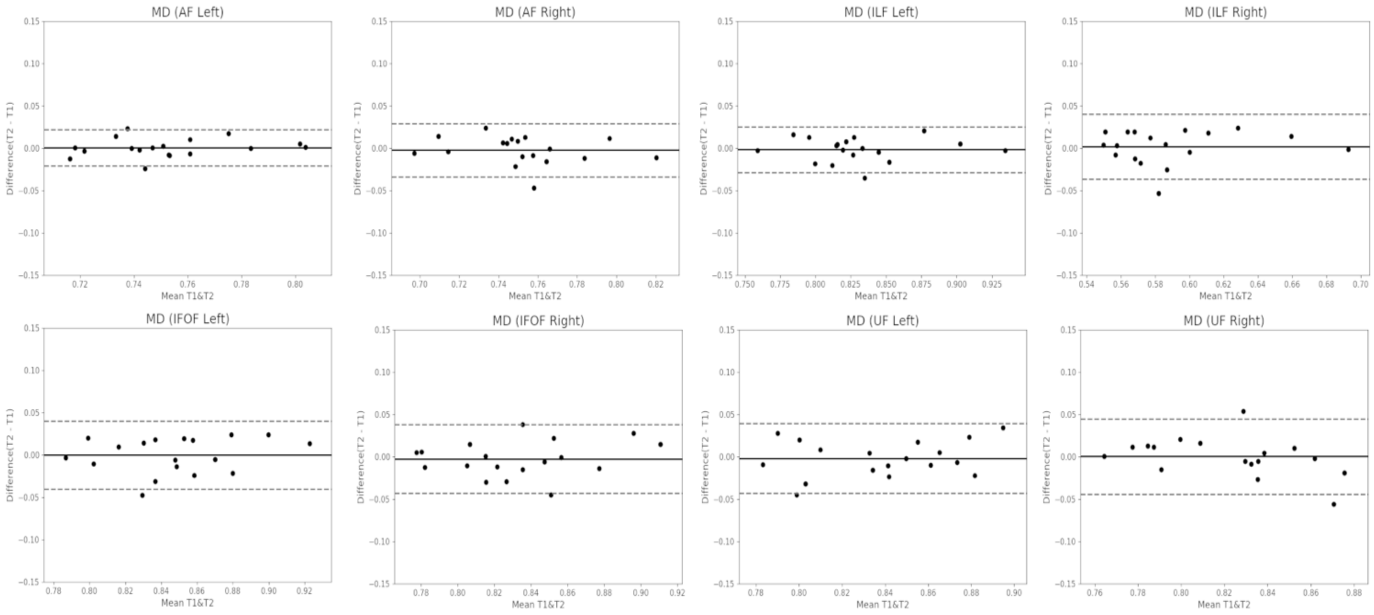

Supplementary Figure 1. Bland-Altman Plots for the MD metric in all four fiber bundles, bilaterally. The Y axis represents the mean difference between the measurements at the two timepoints and the X axis represents the mean of these measures. The upper and lower dashed lines represent the two limits of agreements at ±2 standard-deviations of the mean of differences (i.e. the 95% confidence interval). The solid line represents the mean of the differences between the two timepoints. The dots represent the individual subjects. MD = mean diffusivity; AF = arcuate fasciculus; ILF = inferior longitudinal fasciculus; IFOF = inferior fronto-occipital fasciculus; UF = uncinate fasciculus; T1 = time 1; T2 = time 2.

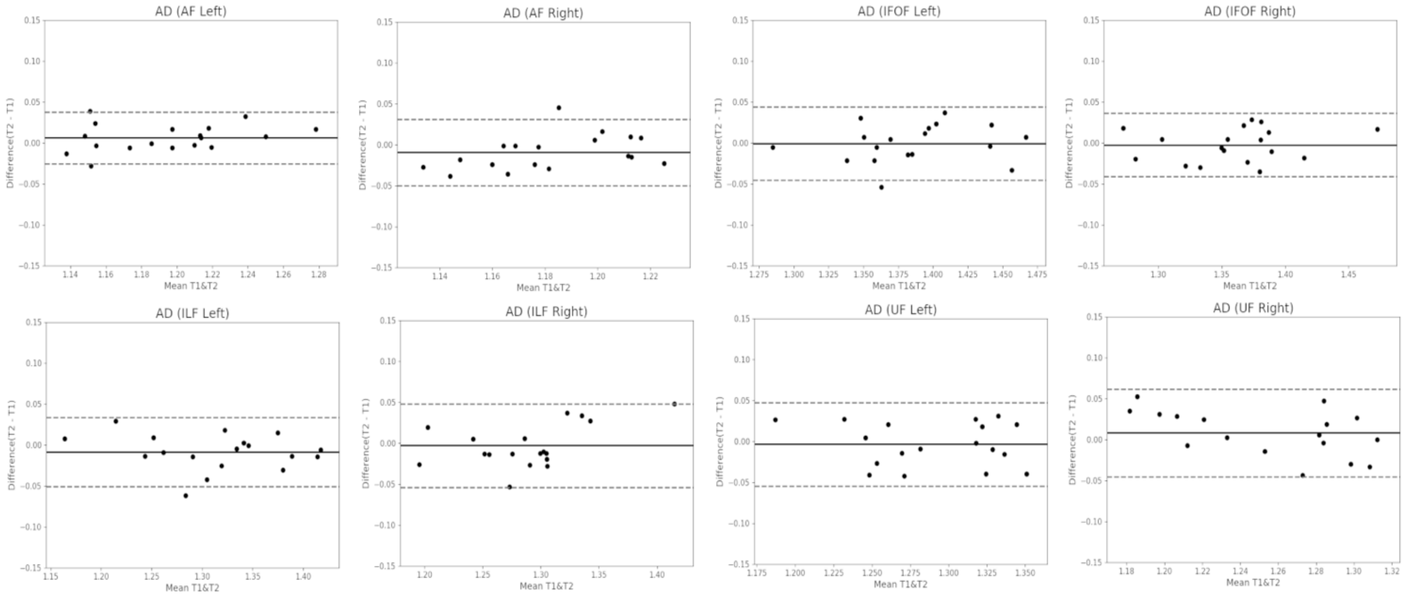

Supplementary figure 2. Bland-Altman Plots for the AD metric in all four fiber bundles, bilaterally. The Y axis represents the mean difference between the measurements at the two timepoints and the X axis represents the mean of these measures. The upper and lower dashed lines represent the two limits of agreements at ±2 standard-deviations of the mean of differences (i.e. the 95% confidence interval). The solid line represents the mean of the differences between the two timepoints. The dots represent the individual subjects. AD = axial diffusivity; AF = arcuate fasciculus; ILF = inferior longitudinal fasciculus; IFOF = inferior fronto-occipital fasciculus; UF = uncinate fasciculus; T1 = time 1; T2 = time 2.

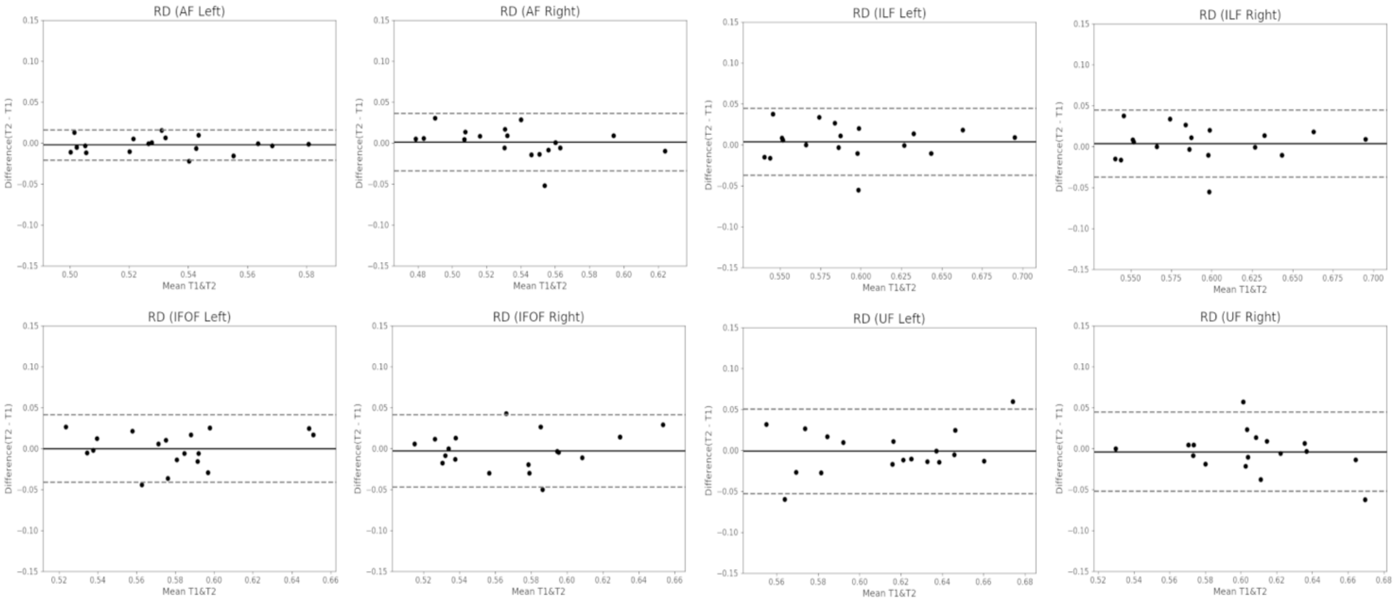

Supplementary figure 3. Bland-Altman Plots for the RD metric in all four fiber bundles, bilaterally. The Y axis represents the mean difference between the measurements at the two timepoints and the X axis represents the mean of these measures. The upper and lower dashed lines represent the two limits of agreements at ±2 standard-deviations of the mean of differences (i.e. the 95% confidence interval). The solid line represents the mean of the differences between the two timepoints. The dots represent the individual subjects. RD = radial diffusivity; AF = arcuate fasciculus; ILF = inferior longitudinal fasciculus; IFOF = inferior fronto-occipital fasciculus; UF = uncinate fasciculus; T1 = time 1; T2 = time 2.

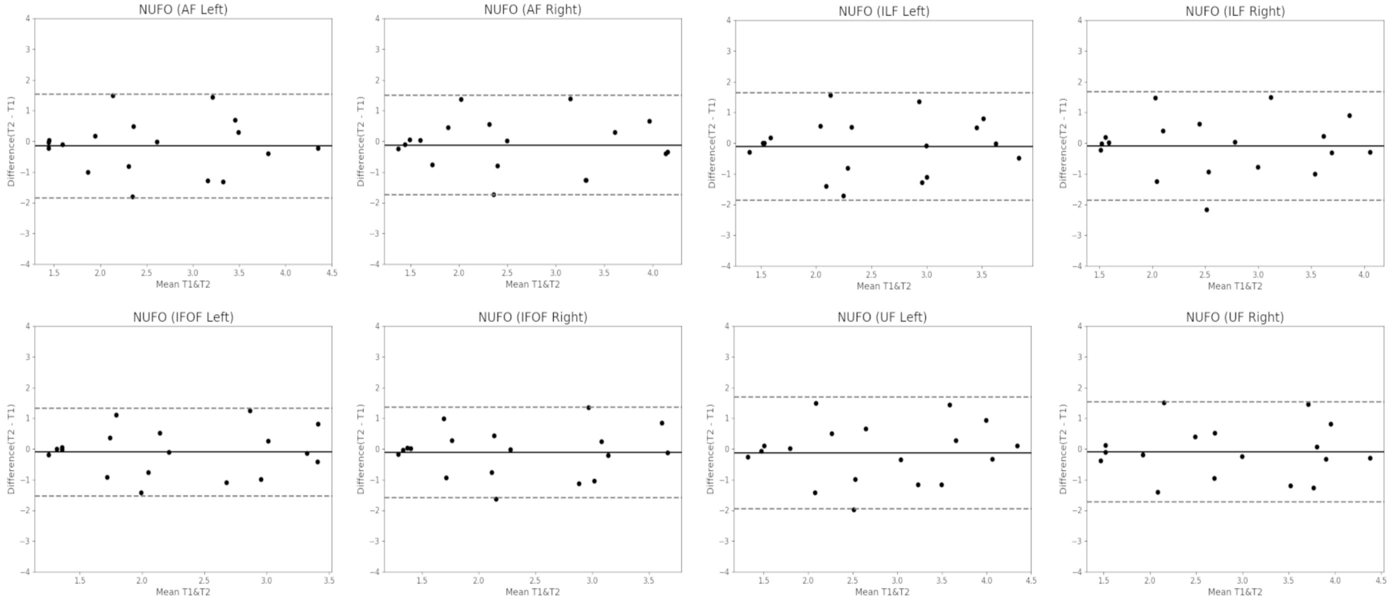

Supplementary figure 4. Bland-Altman Plots for the NuFO measure in all four fiber bundles, bilaterally. The Y axis represents the mean difference between the measurements at the two timepoints and the X axis represents the mean of these measures. The upper and lower dashed lines represent the two limits of agreements at ±2 standard-deviations of the mean of differences (i.e. the 95% confidence interval). The solid line represents the mean of the differences between the two timepoints. The dots represent the individual subjects. NuFO = Number of fiber orientations; AF = arcuate fasciculus; ILF = inferior longitudinal fasciculus; IFOF = inferior fronto-occipital fasciculus; UF = uncinate fasciculus; T1 = time 1; T2 = time 2.

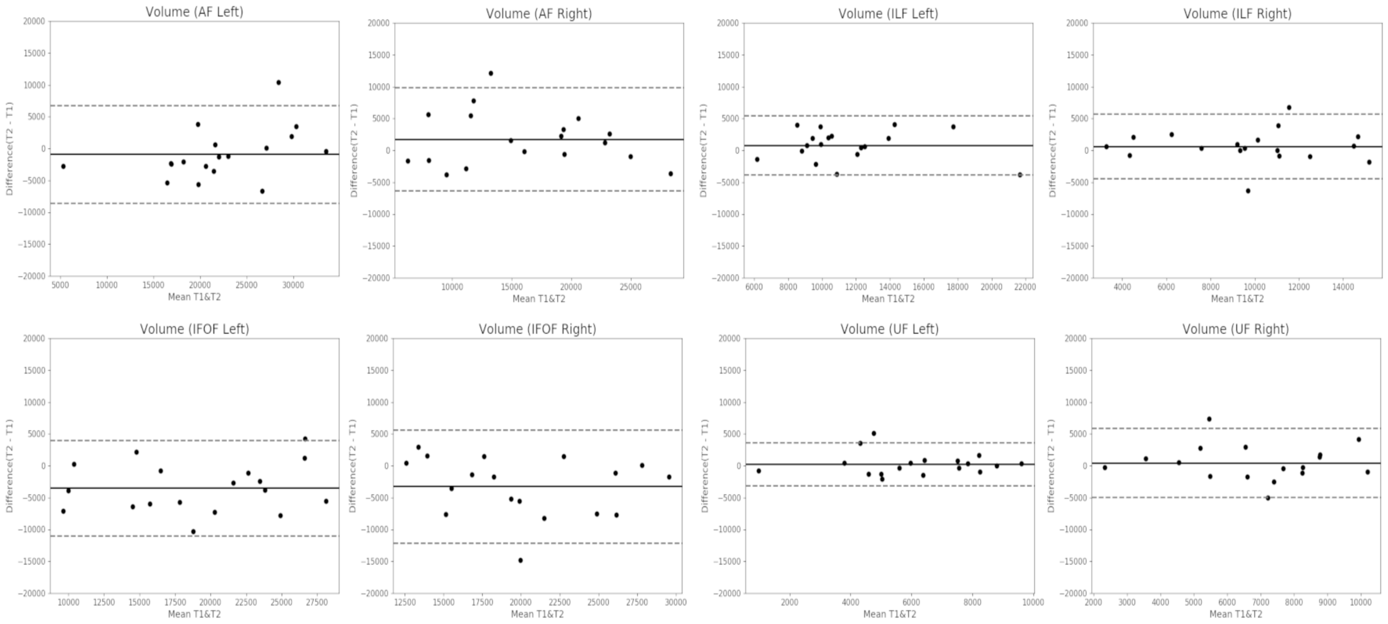

Supplementary figure 5. Bland-Altman Plots for the Volume measure in all four fiber bundles, bilaterally. The Y axis represents the mean difference between the measurements at the two timepoints and the X axis represents the mean of these measures. The upper and lower dashed lines represent the two limits of agreements at ±2 standard-deviations of the mean of differences (i.e. the 95% confidence interval). The solid line represents the mean of the differences between the two timepoints. The dots represent the individual subjects. AF = arcuate fasciculus; ILF = inferior longitudinal fasciculus; IFOF = inferior fronto-occipital fasciculus; UF = uncinate fasciculus; T1 = time 1; T2 = time 2.

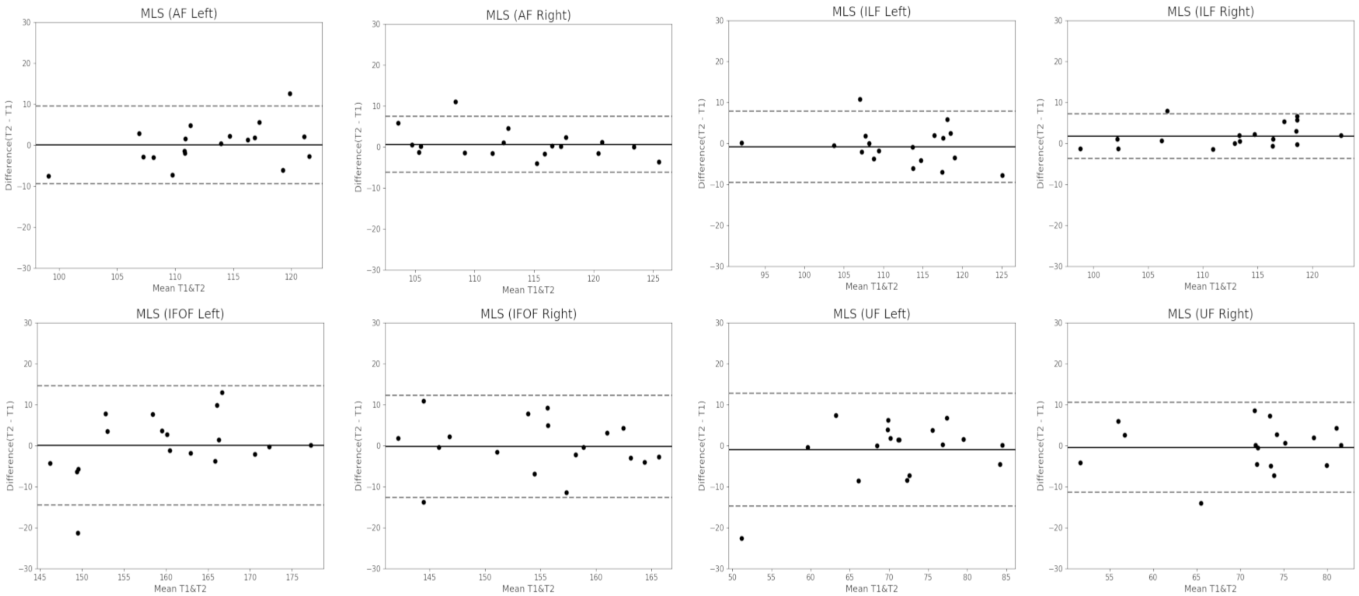

Supplementary figure 6. Bland-Altman Plots for the MLS measure in all four fiber bundles, bilaterally. The Y axis represents the mean difference between the measurements at the two timepoints and the X axis represents the mean of these measures. The upper and lower dashed lines represent the two limits of agreements at ±2 standard-deviations of the mean of differences (i.e. the 95% confidence interval). The solid line represents the mean of the differences between the two timepoints. The dots represent the individual subjects. MLS = mean length of streamlines; AF = arcuate fasciculus; ILF = inferior longitudinal fasciculus; IFOF = inferior fronto-occipital fasciculus; UF = uncinate fasciculus; T1 = time 1; T2 = time 2.
