# Supplementary material for: Test-Retest Reliability of Diffusion Measures Extracted Along White Matter Language Fiber Bundles Using HARDI-Based Tractography
Source: Front Neurosci. 2019 Jan 14;12:1055. doi: 10.3389/fnins.2018.01055 (PMC6339903; doi:10.3389/fnins.2018.01055)
Supplement: Supplementary file 2 [file Table_1.DOCX]

Supplementary Material

Test-retest reliability of diffusion measures extracted along white matter language fiber bundles using HARDI-based tractography

**Mariem Boukadi, Karine Marcotte, Christophe Bedetti, Jean-Christophe Houde, Alex Desautels, Samuel Deslauriers-Gauthier, Marianne Chapleau, Arnaud Boré, Maxime Descoteaux, & Simona M. Brambati^*^**

*** Correspondence:** Simona M. Brambati: simona.maria.brambati@umontreal.ca

# Motion of each participant at each timepoint

| Participant | Time 1 | | Time 2 | |
| --- | --- | --- | --- | --- |
|  | Mean | SD | Mean | SD |
| 1 | 0.06177309 | 0.04426779 | 0.0569417 | 0.03484703 |
| 2 | 0.05257956 | 0.03107867 | 0.05408551 | 0.02999035 |
| 3 | 0.0740012 | 0.04511743 | 0.07651165 | 0.04921323 |
| 4 | 0.08372465 | 0.06744107 | 0.07903643 | 0.04782817 |
| 5 | 0.07697474 | 0.03897778 | 0.04636438 | 0.02777494 |
| 6 | 0.04968429 | 0.03638177 | 0.04530571 | 0.03038225 |
| 7 | 0.06372794 | 0.03709305 | 0.05533408 | 0.02970052 |
| 8 | 0.07719768 | 0.06353363 | 0.05609605 | 0.02513792 |
| 9 | 0.06572525 | 0.03452804 | 0.06984521 | 0.04321858 |
| 10 | 0.04871767 | 0.02983834 | 0.0477965 | 0.029187 |
| 11 | 0.08445199 | 0.05201282 | 0.07557234 | 0.0489541 |
| 12 | 0.04386039 | 0.02260433 | 0.04060202 | 0.02549169 |
| 13 | 0.06665881 | 0.037032 | 0.07046448 | 0.04341005 |
| 14 | 0.07411467 | 0.04105924 | 0.08721011 | 0.05380671 |
| 15 | 0.0562165 | 0.02560989 | 0.06681639 | 0.03486903 |
| 16 | 0.10179909 | 0.05675531 | 0.08396169 | 0.05246243 |
| 17 | 0.06013741 | 0.03774356 | 0.05252436 | 0.03768069 |
| 18 | 0.06618107 | 0.02970252 | 0.09723072 | 0.04166339 |
