# Supplementary material for: Test-Retest Reliability of Diffusion Measures Extracted Along White Matter Language Fiber Bundles Using HARDI-Based Tractography
Source: Front Neurosci. 2019 Jan 14;12:1055. doi: 10.3389/fnins.2018.01055 (PMC6339903; doi:10.3389/fnins.2018.01055)
Supplement: Supplementary file 3 [file Table_2.DOCX]

Supplementary Material

Test-retest reliability of diffusion measures extracted along white matter language fiber bundles using HARDI-based tractography

**Mariem Boukadi, Karine Marcotte, Christophe Bedetti, Jean-Christophe Houde, Alex Desautels, Samuel Deslauriers-Gauthier, Marianne Chapleau, Arnaud Boré, Maxime Descoteaux, & Simona M. Brambati^*^**

*** Correspondence:** Simona M. Brambati: simona.maria.brambati@umontreal.ca

# Modified White Matter Query Language Queries

#Arcuate Fasciculus (AF)

AF.side = (inferior_frontal_gyrus.side or middle_frontal_gyrus.side or precentral.side) and (superiortemporal.side or middletemporal.side) not in hemisphere.opposite not in medial_of(supramarginal.side) not in ILF_final_1.side not in IFOF_final_1.side not in temporalpole.side not in frontalpole.side not in subcortical.side not in rostralmiddlefrontal.side not in lateralorbitofrontal.side not in ec.side not in superiorfrontal.side not in ctx_superiortemporal.side not in ctx_insula.side

#Inferior Longitudinal Faciculus (ILF)

ILF.side= only(temporal.side and occipital.side) and anterior_of(hippocampus.side) not in parahippocampal.side not in ctx_lingual.side not in ctx_lateraloccipital.side not in ctx_cuneus.side not in ctx_pericalcarine.side

#Inferior Fronto-Occipital Fasciculus (IFOF)

IFOF.side= endpoints_in(orbitofrontalgyrus.side or inferior_frontal_gyrus.side) and endpoints_in(occipital.side) and temporal.side and insula.side not in inferiorparietal.side not in hemisphere.opposite

#Uncinate Fasciculus (UF)

UF.side= insula.side and endpoints_in(orbitofrontalgyrus.side) and endpoints_in(temporal_anterior_section.side) not in posterior_of(putamen.side) not in centrum_semiovale.side not in superiorfrontal.side not in postcentral.side not in precentral.side
